# A Clinico-Morphological Study of Acrochordons and the Association With Diabetes Mellitus

**DOI:** 10.7759/cureus.78549

**Published:** 2025-02-05

**Authors:** Bebisha Joseph Chandran, Pradeep Nair S

**Affiliations:** 1 Dermatology, KIMSHEALTH Medical Centre, Doha, QAT; 2 Dermatology and Venereology, Government T. D. (Thirumala Devaswom) Medical College Alappuzha, Alappuzha, IND

**Keywords:** acrochordons, clinical, diabetes, insulin resistance, skintags

## Abstract

Background

Acrochordons, otherwise called soft fibromas or skin tags, are small, soft, pedunculated protrusions occurring mainly on the neck and major flexures. They are being investigated as one of the cutaneous markers of diabetes mellitus (DM). The objective of the study was to analyze the clinico-morphological types of acrochordons and to study the association of acrochordons with DM.

Materials and methods

A descriptive comparative study was conducted in the dermatology outpatient department of a tertiary care hospital in South India over a period of one year. It included 150 cases with acrochordons and a comparative group with age- and sex-matched individuals without acrochordons. All the clinically confirmed cases and comparative groups were subjected to detailed history, clinical examination, and blood and urine tests, and the details were noted in the standard proforma. Statistical analysis was done using Pearson’s Chi-squared test.

Results

Statistical analysis revealed a statistically significant relationship between skin tags and DM (p = 0.0073). Also, there was a significant association between the duration of skin tags and the duration of DM (p= 0.000). The odds ratio was found to be 1.89, which implies that there is a 1.8 times greater risk of an individual with ST developing DM when compared with an individual without a skin tag.

Conclusion

Skin tags can be considered as an early marker of impaired carbohydrate metabolism and DM.

## Introduction

Acrochordon is a common, benign lesion composed of loose fibrous tissue and occurring mainly on the neck, axilla, and eyelids and less commonly in the trunk and groins [1]. It is synonymous with skin tags, soft fibromas, and fibroepithelial polyps. The incidence of acrochordons is around 46% in adults more than 40 years of age. There is no difference in incidence between male and female individuals [2]. They often develop in areas of skin friction [3]. They have been reported to be associated with many diseases including acromegaly, symptomatic intestinal polyps, dyslipidaemia, obesity, diabetes mellitus (DM), atherosclerosis, and various syndromes including polycystic ovary syndrome, Birt-Hogg-Dubé syndrome, and Cowden’s syndrome [2].

Acrochordons are being investigated as one of the cutaneous markers of DM. As India is emerging as the diabetes capital of the world, a study of acrochordon and its association with DM may help in the establishment of acrochordon as a cutaneous marker of DM.

## Materials and methods

This was a descriptive comparative study conducted in the Department of Dermatology and Venereology, Government Medical College Hospital, Thiruvananthapuram, Kerala, India, over a period of one year. The study was approved by the Human Ethics Committee, Medical College, Thiruvananthapuram (approval number: 07/21/2012MCT).

Inclusion and exclusion criteria

A total of 150 clinically diagnosed cases of acrochordons were included in the study. Patient selection was by consecutive sampling. Patients not consenting to the study were excluded. Age and sex-matched individuals without acrochordons were taken as the comparative group after consent.

Data collection

After informed consent, clinically diagnosed cases of acrochordons were subjected to detailed history and dermatological examination. The duration, gender, age and occupation were the main demographic data collected. A past history of DM was questioned in all the cases. Family history of acrochordons and DM were also taken into detail. The size, colour, number of lesions, and distribution were the main clinical features noted. All the clinical details collected were recorded in the standard preformatted proforma.

Investigations

All the subjects were subjected to fasting blood sugar (FBS) and post-prandial blood sugar testing (PPBS). An FBS level of >126 mg% and a PPBS level of >200 mg% were the parameters to diagnose DM, as per the American Diabetic Association (ADA) guidelines. Urine tests done were sugar, albumin and deposits. The comparative group was also subjected to FBS/PPBS and routine urine examination after consent.

Statistical analysis

The data collected were analysed in terms of mean, frequency, and percentage. The Chi-squared test was applied to note if there was any statistical significance between the patients with acrochordons and the comparative group, and a ‘P’ value less than 0.05 was considered to be statistically significant. The odds ratio (OR) was also calculated.

## Results

**Figure 1:**
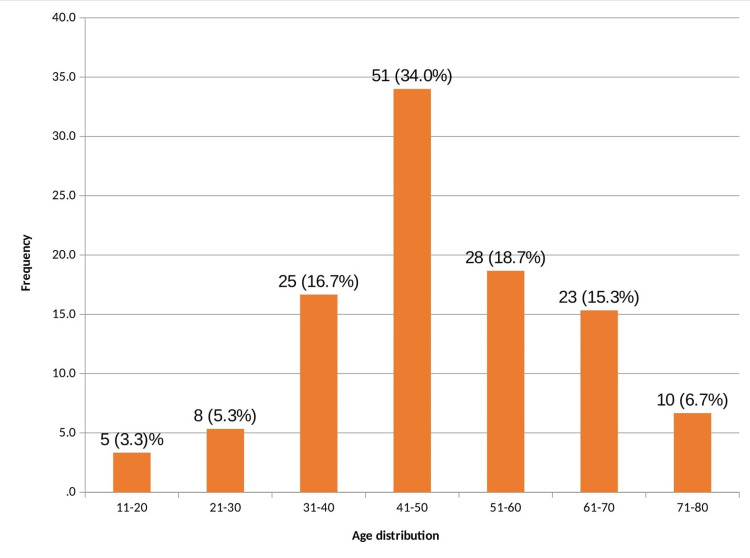
Age distribution of the patients (N=150)

Among the 150 cases included in the study, 51 patients (34%) were in the age group of 41-50 years; the youngest patient was a 14-year-old female and the oldest patient was an 80-year-old male. The mean age was 48.33 years (Figure 1).

A near-equal gender distribution was observed with a male-to-female ratio of 0.97:1. The duration of acrochordons ranged from three months to 30 years, the mean duration being 8.72 years. Statistical analysis revealed a significant association between the duration of skin lesions and the duration of DM (p = 0.000).

DM was the only associated illness in 33 patients. DM along with hypertension was noted in 24 patients, and DM, hypertension, and dyslipidaemia were present in four of them. To sum up, there were 61 patients with a history of DM. Family history of skin tags was present in 67 patients (44.7%). No significant association was observed between family history of skin tags and DM (p = 0.523). A family history of DM was observed in 65 out of the 150 cases in the study (43.3%). There was no statistically significant association between patients with skin tags and family history of DM (p = 0.1).

Of the 61 patients with DM, 34 of them had a duration of DM between one and five years (22.7%). The mean duration of DM was 6.26 years. The majority of the DM patients did not have any evident complications; three of them had diabetic nephropathy and one had diabetic retinopathy. Among 150 cases, 18 (12%) were found to have increased blood pressure, which was found to be statistically insignificant in DM patients (p = 0.65). Most of the patients weighed 61-70 kgs.

**Figure 2:**
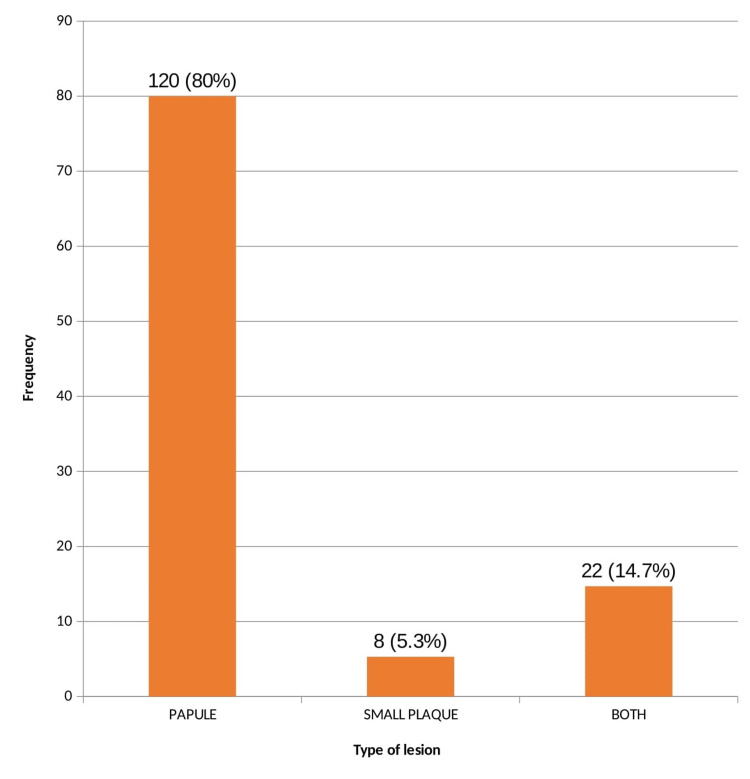
Types of skin lesions

Of the 74 DM patients with skin tags, 66 patients weighed more than 60 kg. On analysis, there was no statistical significance (p=0.06). The majority of the cases had papules as the primary lesions (n=120, 80%). Both papules and small plaques were present in 22 patients (14.7%) (Figure 2). There was no significant association between the type of skin lesion and patients with DM (p = 0.31).

**Figure 3:**
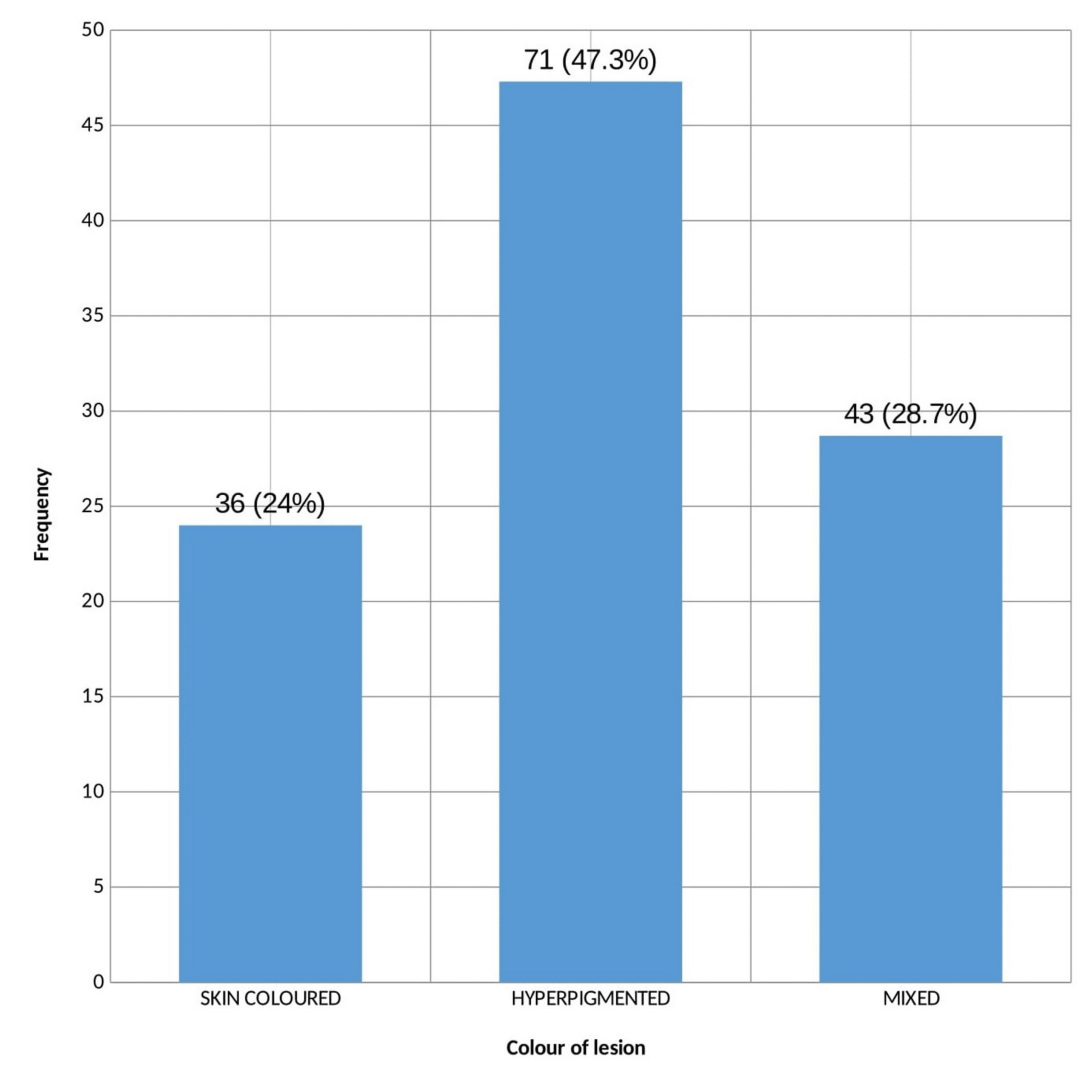
Colour of lesions

Hyperpigmented skin tags were present in 71 cases (47.3%) followed by mixed type in 43 patients (28.7%) (Figure 3). No significant association was noted between the colour of skin lesions and DM (p = 0.941).

Pedunculated skin tags were present in 72 cases (48%). Fifty-one patients (34%) had sessile lesions whereas 27 patients (18%) had both types of lesions. There is no statistical significance between the peduncle of the skin lesion and DM (p = 0.47).

**Figure 4:**
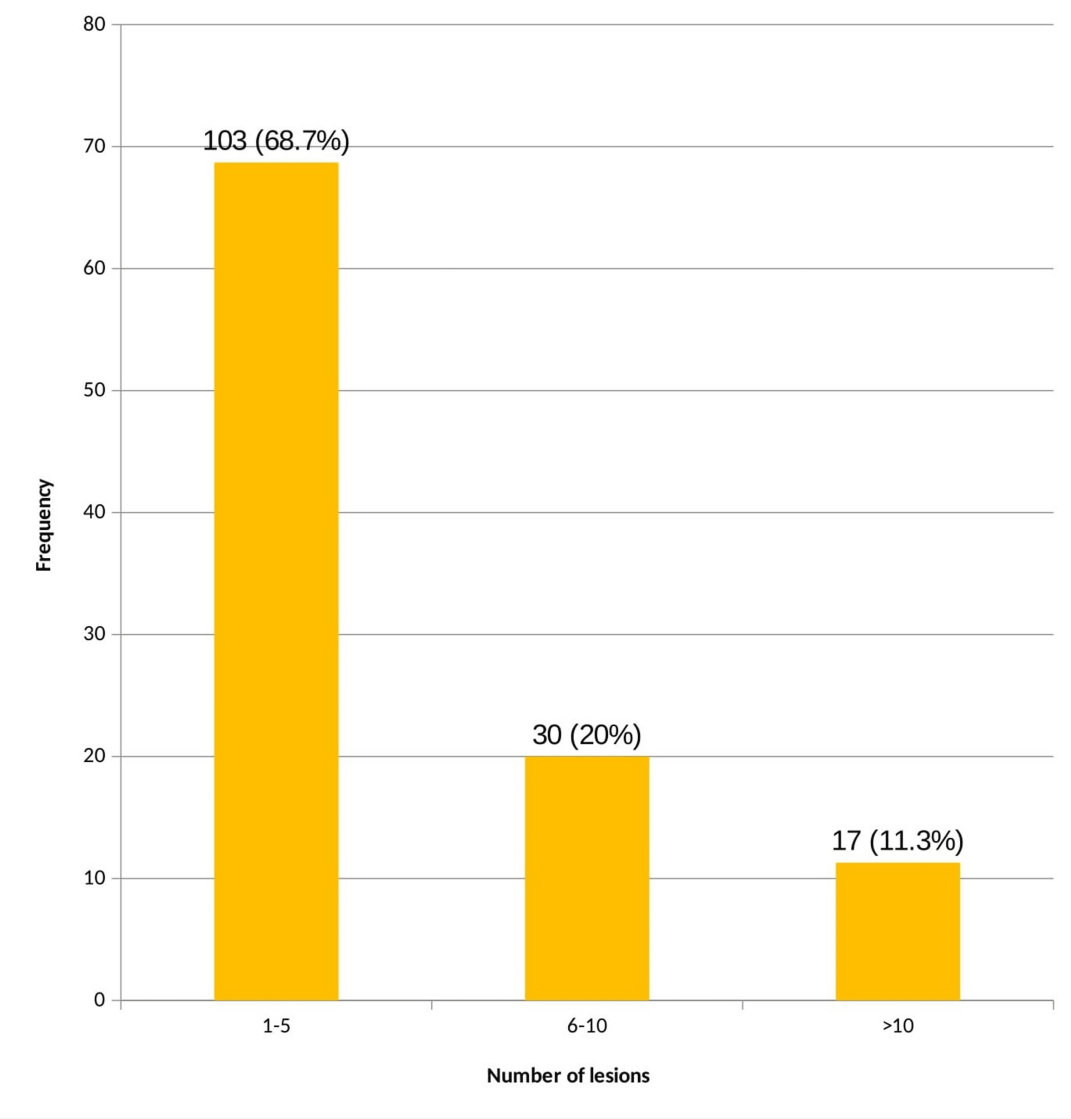
Number of lesions

Total number of lesions varied from one to 30. The majority of the cases (n=103; 68.7%) had one to five lesions. Mean number of lesions is six. There was no significant relationship between the number of skin lesions and the presence of DM (p = 0.36) (Figure 4).

**Figure 5:**
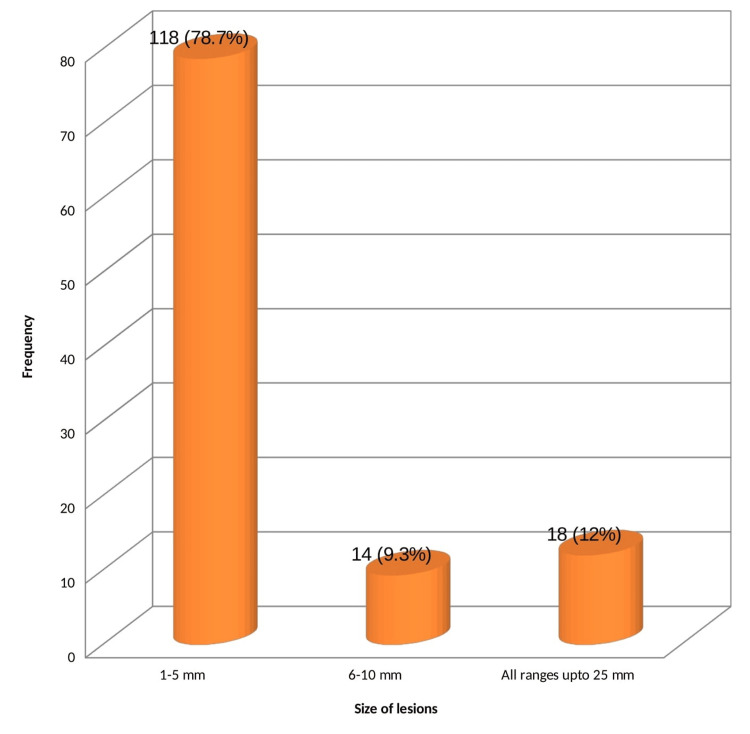
Size of lesions

A total of 118 patients had lesions, sizes ranging from 1-5 mm (78.7%). The size of lesions varied from 1 mm to 25 mm. The mean size of lesions was 5.21 cm. Eighteen patients had lesions of all sizes ranging from 1 mm to 25 mm and 11 of them had DM. However, there was no statistically significant association with DM (p = 0.42) (Figure 5).

**Figure 6:**
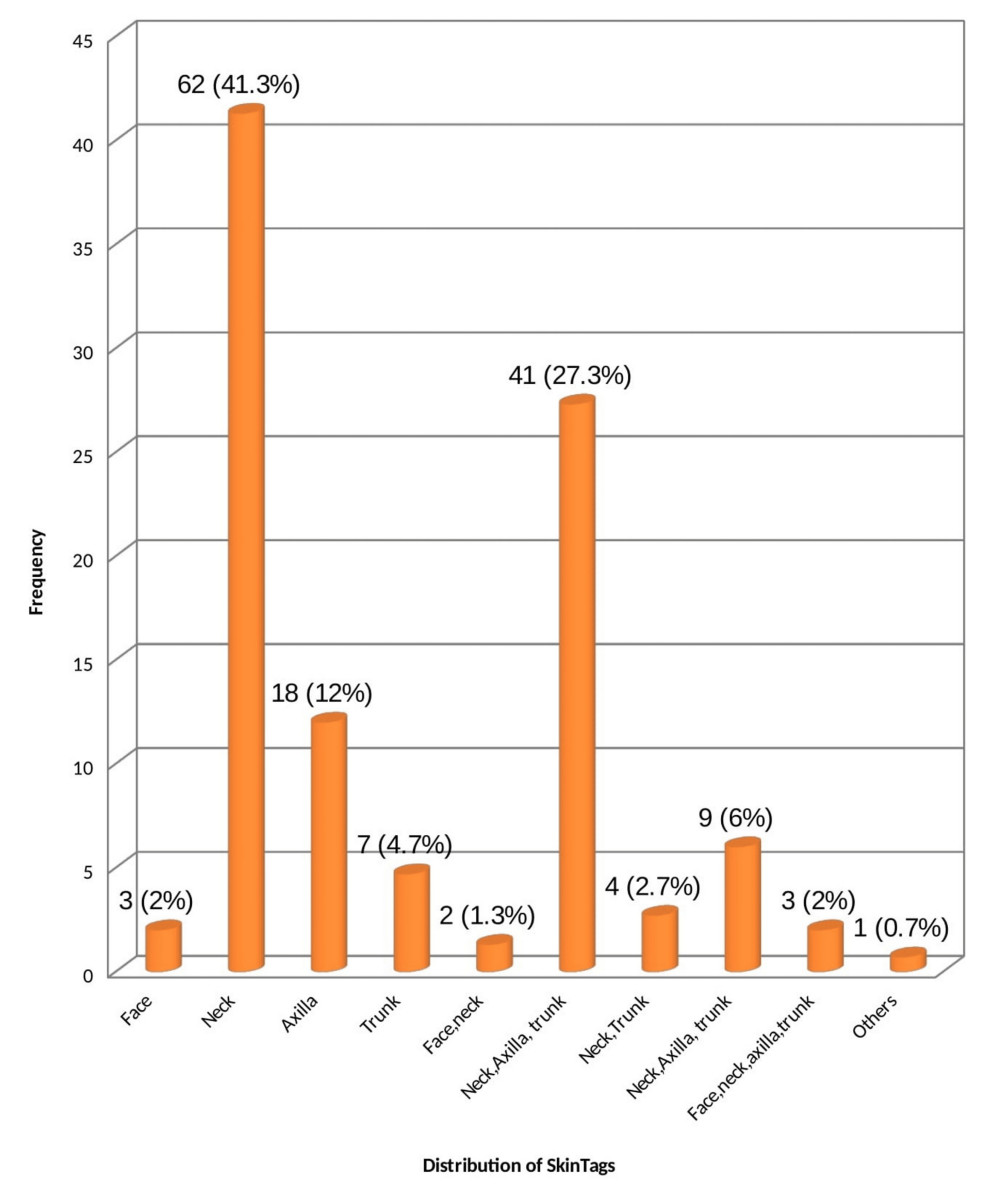
Distribution of skin tags

Skin tags were distributed mainly on the neck (n=62, 41.3%) followed by the neck and axilla in 41 patients (27.3%). Only the axilla was involved in 18 patients (12%). There was no specific distribution noted among the DM patients and it did not carry any statistical significance (p = 0.32) (Figure 6).

Dermatosis papulosa nigra was observed as an associated skin lesion in 55 cases followed by seborrheic keratosis in 34 of them (Table 1). Tables 2, 3 give summarised results of the demographic and clinical details of the participants.

**Table 1:** Associated skin lesions in the patients (N=150)

Associated skin lesions	Number of patients
Dermatosis Papulosa Nigra	55
Hirsutism	4
Seborrhoeic Keratoses	34
Acanthosis Nigricans	23
Idiopathic Guttate Hypomelanosis	3
Psoriasis	12
No other skin lesions	37

**Table 2:** Summarised results of study: demographic details OHAS: oral hypoglycemic agents

Demographic features	Subcategories	Frequency (n)	Percentage (%)
Age group (years)	11-20	5	3.3
	21-30	8	5.3
	31-40	25	16.7
	41-50	51	34.0
	51-60	28	18.7
	61-70	23	15.3
	71-80	10	6.7
Gender	Male	74	49.3
	Female	76	50.7
Marital status	Married	133	88.7
	Unmarried	17	11.3
Occupation	Professional	5	3.3
	Skilled	14	9.3
	Semiskilled	21	14.0
	Unskilled	25	16.7
	Student	6	4.0
	Unemployed	22	14.7
	Homemaker	57	38.0
Duration of acrochordons (years)	<1	7	4.7
	1-5	59	39.3
	6-10	43	28.7
	11-15	15	10.0
	16-20	9	6.0
	>20	17	11.3
Other illness associated	No illness	76	50.7
	Diabetes mellitus	33	22.0
	Hypertension	6	4.0
	Dyslipidaemia	3	2.0
	Diabetes + hypertension	24	16.0
	Diabetes +hypertension + dyslipidaemia	4	2.7
	Others (bronchial asthma, seizures)	4	2.7
Family history of skin tags	Present	67	44.7
	Absent	83	55.3
Family history of diabetes	Present	65	43.3
	Absent	85	56.7
Duration of diabetes (years)	<1	7	4.7
	1-5	34	22.7
	6-10	6	4.0
	11-15	8	5.3
	16-20	3	2.0
	>20	3	2.0
Treatment for diabetes	Diet	6	4.0
	OHAS	47	31.3
	Insulin	5	3.3
	All	3	2.0
Complications of diabetes mellitus	Present	4	2.7
	Absent	57	38.0

**Table 3:** Summarised clinical features

Clinical features	Subcategories	Frequency (n)	Percentage (%)
Weight	<40 Kg	2	1.3
	41-50 Kg	5	3.3
	51-60 Kg	23	15.3
	61-70 Kg	65	43.3
	71-80 Kg	31	20.7
	>81 Kg	24	16.0
Primary lesion	Papule	120	80.0
	Small plaque	8	5.3
	Both	22	14.7
Color of lesion	Skin coloured	36	24.0
	Hyperpigmented	71	47.3
	Mixed	43	28.7
Peduncle	Pedunculated	72	48.0
	Sessile	51	34.0
	Both	27	18.0
Number of lesions	1-5	103	68.7
	6-10	30	20.0
	>10	17	11.3
Size of lesions	1-5 mm	118	78.7
	6-10 mm	14	9.3
	Up to 25 mm	18	12.0
Distribution of skin tags	Face	3	2.0
	Neck	62	41.3
	Axilla	18	12.0
	Trunk	7	4.7
	Face, neck	2	1.3
	Neck, axilla	41	27.3
	Neck, trunk	4	2.7
	Neck, axilla, trunk	9	6.0
	Face, neck, axilla, trunk	3	2.0
	Others (thigh)	1	0.7

**Figure 7:**
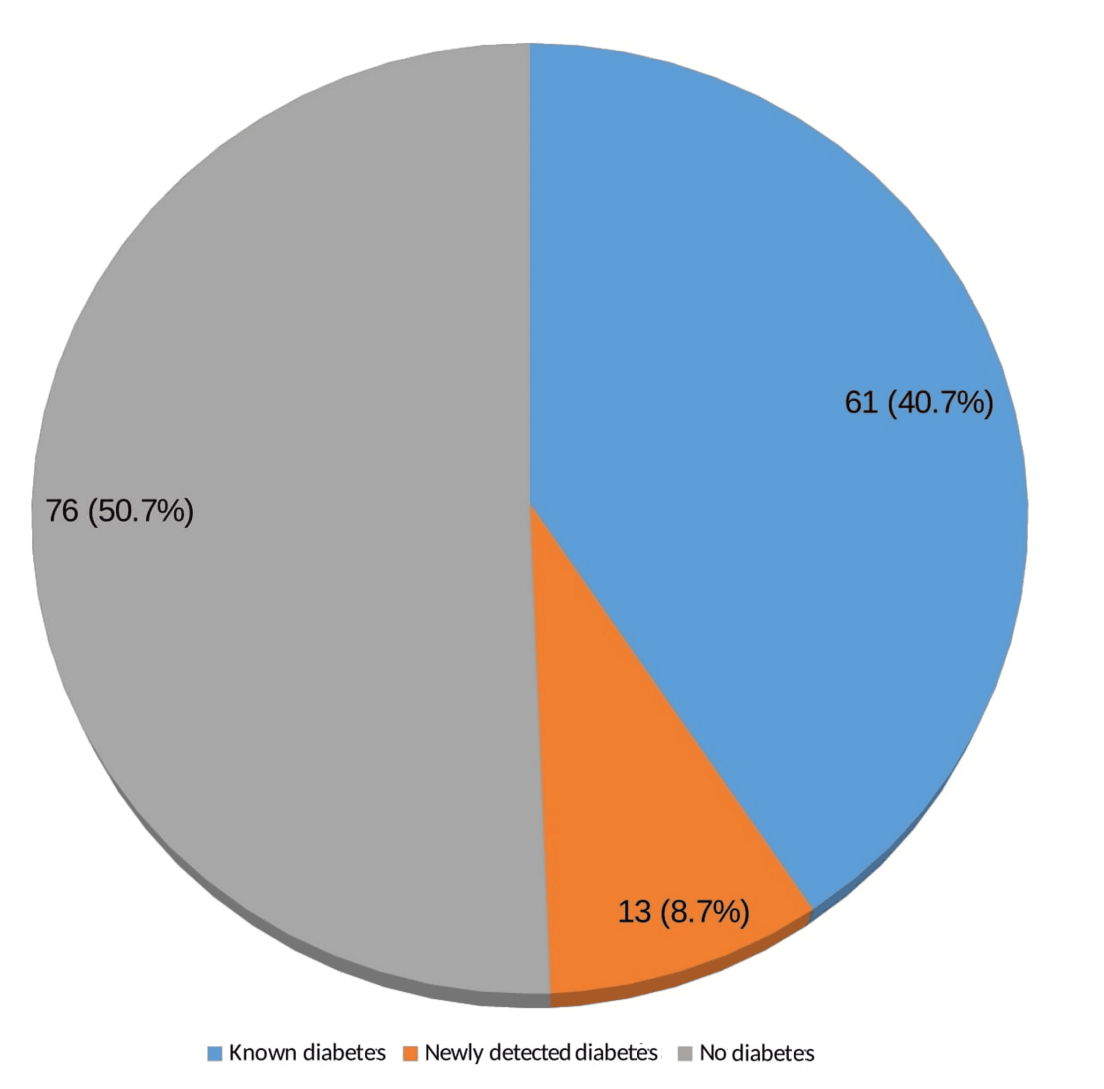
Distribution of patients with skin tags according to presence of diabetes mellitus (N=150) Data presented as n (%)

Urine sugar was positive in 12 cases (8%) and all of them were patients with DM. Increased blood sugar levels were present in 29 known DM patients and 32 known DM patients on treatment had normal blood sugar levels. A total of 13 patients without a history of DM were found to have increased blood sugar. To sum up, 74 (49.3%) patients in our study had gas DM (Figure 7).

**Figure 8:**
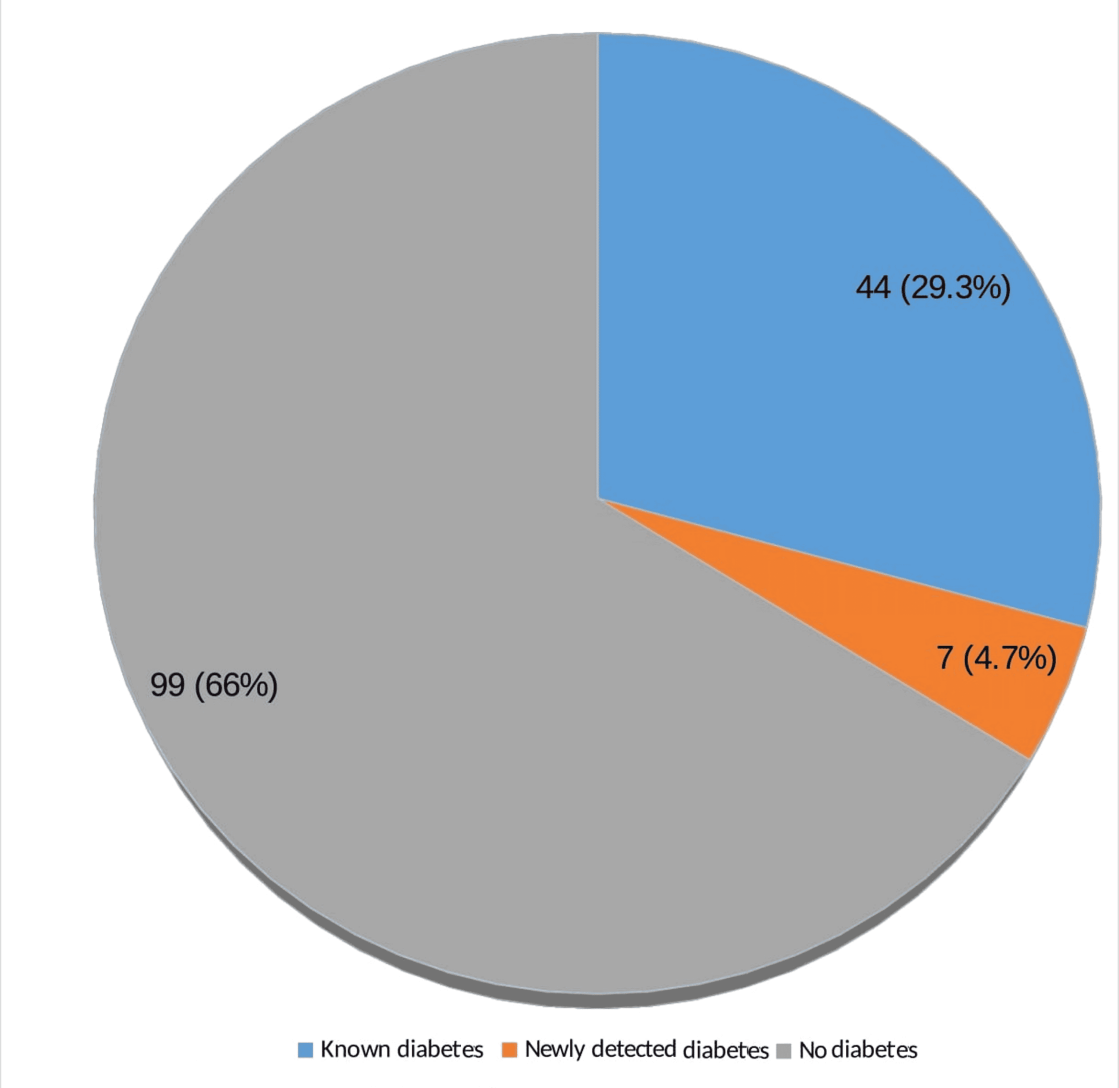
Distribution of the comparative group (without skin tags) according to the presence of diabetes mellitus (N=150) Data presented as n (%)

Among the 150 age- and sex-matched individuals included in the comparative group, 44 were known cases of DM of whom only 27 had increased blood sugar levels. There were seven newly detected with DM. In total, 51 (34%) persons had DM in the comparative group (Figure 8).

Thus, it was found that 74 (49.3%) patients with skin tags have DM whereas 51 (34%) individuals in the comparative group (without skin tags) had DM. On statistical analysis, it was found that there was a statistically significant relationship between skin tags and DM (p = 0.0073) as compared to the comparative group. The OR was found to be 1.89 which implies that there is a 1.8 times greater risk in an individual with skin tag to develop DM when compared with an individual without skin tag.

## Discussion

Skin tags are small soft benign skin tumours that are commonly found in the general population. Though it is a common condition, it is found to be associated with certain systemic diseases such as DM, metabolic syndrome, symptomatic intestinal polyps, dyslipidaemia, obesity, and polycystic ovarian syndrome. Köseoğlu et al. suggested that hyperinsulinemia and insulin resistance play a role in the development of acrochordon at the tissue receptor level, and could be mediated by IGF-1 [4]. India has one of the highest prevalence of type 2 DM (T2DM) in the world. Recent studies in India show an increasing trend in T2DM in adolescents and children [5]. Many studies have been conducted worldwide to assess the relationship between skin tags and DM.

The mean age of the study population in the current study was 48.33 years ranging from 14 years to 80 years. Also, 74.7% were more than 40 years of age. In a study done by Sherin et al., more than 60% of patients belonged to the age group of 31-50 years [1]. Only 3.3% had skin tags in the age group of 11-20 years and there were no patients below 10 years of age. This is in agreement with the statement that there is an increased incidence of skin tags with increasing age reaching 59% at 70 years of age [2].

There were 74 (49.3%) males and 76 (50.7) females in this study. In a similar study done by Senel et al., 54.54% of the participants were female and 45.45% were male [6].

The duration of skin tags ranged from three months to 30 years. Many patients were not able to recollect the exact duration of the skin lesion because of the benign nature of the lesion, decreased cosmetic concern, and common occurrence of skin tags in the general population. History of DM was present in 40.7% of the cases. In a similar study conducted by Rasi et al., 16 patients (15.38%) had a history of DM [7]. A family history of ST was present in 44.7% of the patients in the present study. This was in close agreement with the study by Rasi et al. [7], and Gönülal et al. [8]. Although a familial component has been reported in the literature, the genetic segregation pattern is yet to be identified [2]. However, there was no statistically significant association between family history of skin tags and DM in this study (p = 0.523). A family history of DM was observed in 43.3% of the patients. According to Rasi et al., a positive family history of DM was found in 30.76% of their patients [7]. In the current study, 34 out of the 61 patients had a history of DM for more than 20 years. Statistical analysis in the present study showed that there was a significant association between the duration of skin tags and the duration of DM (p = 0.000), which has not been reported in similar studies so far.

In 16% of the cases, weight was more than 80 kg. Of the 74 DM patients with skin tags, 66 patients weighed more than 60 kg. In a study done by Sari et al., 53.9% and 33.6% of patients with skin tags were overweight and obese, respectively [9]. Greene et al. conducted a study in the paediatric population and concluded that skin tags are associated with obesity and signs of metabolic syndrome [10].

Among 150 cases, 12% were found to have increased blood pressure which was found to be statistically insignificant in DM patients with acrochordons (p = 0.65). In a study conducted by Shah et al., significantly higher mean systolic and diastolic arterial pressure values were detected in the patient group [11].

Most of the patients had papule as the primary lesion (80%) in the present study while hyperpigmented skin tags were present in 47.3% followed by mixed type in 28.7%. In a similar study done in Varanasi by Agarwal et al., of a total of 118 patients with skin tags, skin-coloured skin tags were present in 69 patients and no correlation was observed between the characteristics of skin tags and abnormal glucose tolerance [12]. According to the literature, the colour of skin tags is probably related to constitutional pigmentation [2]. This explains the increased incidence of hyperpigmented skin tags among this study population

The majority of the cases (68.7%) had a number of lesions between one to five, and 11.3% of the patients had more than 10 lesions. Rasi et al., in their study, concluded that patients with more than 30 skin tags had a significantly higher incidence of impaired carbohydrate metabolism than patients who had less than 30 skin tags [7].

In the present study, skin tags were distributed mainly on the neck in 41.3% of the patients and 39.3% of the patients had multiple-site involvement. Multiple sites were involved in 36% of the patients in a study by Bhargava et al. They concluded that if skin tags are multiple (more than three) and involve multiple sites, they can be taken as a marker for DM [13]. There was no specific distribution pattern, number, or morphological peculiarities of skin tags observed among the DM patients. This is in concordance with the studies done by Demir et al. [14], Sari et al. [9], and Senel et al. [6].

The most common skin lesion observed in association with skin tags was dermatosis papulosa nigra, which was present in 55 out of the 150 cases of the present study. Seborrheic keratosis was present in 34 patients in the present study. Skin tags are frequently associated with seborrheic keratosis and they both represent proliferative skin conditions where growth factors are involved [2]. Both conditions are commonly found in old age. Acanthosis nigricans was present in 23 patients in this study. In a similar study by Rasi et al., acanthosis nigricans was present in six patients (5.76%) with skin tags [7]. According to the literature, acanthosis nigricans with skin tags is said to have more significance than skin tags alone in terms of impaired carbohydrate metabolism [15].

Among the 150 patients with ST, 74 had DM in this study contributing to 49.3% and 51 persons had DM in the comparative group (34%). These findings are in close agreement with the study conducted by Agarwal et al., where DM was present in 40.6% of the patients with skin tags [12]. In a study conducted by Shah et al., 52% of patients and 10% of controls were diagnosed with overt DM and this difference was statistically significant [11]. Karki et al.’s study confirms this finding [16].

On statistical analysis, it was found that there is a statistically significant relationship between skin tags and DM in this study (p = 0.0073). The odds ratio was found to be 1.89 which implied that there is a 1.8 times greater risk in an individual with skin tags to develop DM when compared with an individual without skin tags. This is in agreement with Agarwal et al., who found that the incidence of glucose intolerance in patients with skin tags is at least three times higher than that recorded in the general population, which suggests that there is an association between skin tags and glucose intolerance [12]. Rasi et al. [7], Karki et al. [16], and Demir et al. [14], also supported this finding.

Limitations of study

The sample size was relatively small as the prevalence of acrochordons is very high in the general population.

## Conclusions

This study has shown there is a statistically significant association between acrochordons and DM. There is a significant relationship between the duration of the onset of DM and the duration of acrochordons. Hence, patients with acrochordons should be evaluated at the earliest for the presence of impaired carbohydrate metabolism and DM. Skin tags can be considered a marker of impaired carbohydrate metabolism and prediabetes. Blood glucose levels should be monitored at regular intervals in patients with skin tags. This will help in the prevention of complications of DM by advocating lifestyle changes and proper medications at the earliest.
